# tDCS over the primary motor cortex contralateral to the trained hand enhances cross-limb transfer in older adults

**DOI:** 10.3389/fnagi.2022.935781

**Published:** 2022-09-20

**Authors:** Elisabeth Kaminski, Tom Maudrich, Pauline Bassler, Madeleine Ordnung, Arno Villringer, Patrick Ragert

**Affiliations:** ^1^Department of Movement Neuroscience, Faculty of Sport Science, Leipzig University, Leipzig, Germany; ^2^Department of Neurology, Max Planck Institute for Human Cognitive and Brain Sciences, Leipzig, Germany; ^3^Pediatric Epidemiology, Department of Pediatrics, Medical Faculty, Leipzig University, Leipzig, Germany; ^4^Berlin School of Mind and Brain, Humboldt-Universität zu Berlin, Berlin, Germany; ^5^Charité-Universitätsmedizin Berlin, Humboldt-Universität zu Berlin, Berlin, Germany

**Keywords:** manual dexterity, grooved pegboard task, transcranial direct current stimulation, aging, cross-limb transfer

## Abstract

Transferring a unimanual motor skill to the untrained hand, a phenomenon known as cross-limb transfer, was shown to deteriorate as a function of age. While transcranial direct current stimulation (tDCS) ipsilateral to the trained hand facilitated cross-limb transfer in older adults, little is known about the contribution of the contralateral hemisphere to cross-limb transfer. In the present study, we investigated whether tDCS facilitates cross-limb transfer in older adults when applied over the motor cortex (M1) contralateral to the trained hand. Furthermore, the study aimed at investigating short-term recovery of tDCS-associated cross-limb transfer. In a randomized, double-blinded, sham-controlled setting, 30 older adults (67.0 ± 4.6 years, 15 female) performed a short grooved-pegboard training using their left hand, while anodal (a-tDCS) or sham-tDCS (s-tDCS) was applied over right M1 for 20 min. Left (LH_*trained*_) - and right-hand (RH_*untrained*_) performance was tested before and after training and in three recovery measures 15, 30 and 45 min after training. LH_*trained*_ performance improved during both a-tDCS and s-tDCS and improvements persisted during recovery measures for at least 45 min. RH_*untrained*_ performance improved only following a-tDCS but not after s-tDCS and outlasted the stimulation period for at least 45 min. Together, these data indicate that tDCS over the M1 contralateral to the trained limb is capable of enhancing cross-limb transfer in older adults, thus showing that cross-limb transfer is mediated not only by increased bi-hemispheric activation.

## Introduction

During the aging process, the capability of learning novel motor skills declines (Voelcker-Rehage, 2008; Roig et al., 2014; VanSwearingen and Studenski, 2014; Cirillo, 2021). Additionally, motor performance becomes less reliable and a series of basic motor functions such as maximal strength and force steadiness but also performance of complex fine motor tasks decreases as a function of age (Voelcker-Rehage, 2008; Hunter et al., 2016). Motor dexterity is one of the key functions for the ability of independent living (Ranganathan et al., 2001a; Choi et al., 2017) and is defined as the ability to perform coordinated finger movements in order to grasp and manipulate small objects (Makofske, 2011). Research showed that for complex fine motor tasks, a stable level of motor dexterity is maintained until around 70–75 years (Hoogendam et al., 2014), while after this critical age period, performance declines rapidly (Smith et al., 1999). In simpler fine motor tasks, linear effects of age on manual dexterity have been reported, starting around the age of 65 years (Ranganathan et al., 2001a; Adler et al., 2002; Hoogendam et al., 2014). Among others, age-related impairments in manual dexterity were found regarding sensory functioning (Cole et al., 1999; Goble et al., 2009) and the ability to grasp small objects (Sears and Chung, 2010; Friedman et al., 2011). One underlying factor for age-related impairments is an age-related reorganization of the brain on a functional and structural level (Peters, 2006; Zapparoli et al., 2022). On the structural level, this age-related process manifests itself in a loss of fibers in gray matter (Sowell et al., 2003) but also white matter alterations (Guttmann et al., 1998; Pantoni, 2010) as well as chemical changes, such as a decrease of dopamine and serotonin receptors (Iyo and Yamasaki, 1993; Seidler et al., 2010). On the functional level, aging is associated with a reduction of task-based hemispheric asymmetry (Cabeza, 2002), which was found to be a compensatory mechanism associated with improved performance in some studies (Cabeza et al., 2018) while other findings indicate a rather non-specific mechanism of dedifferentiation (Cassady et al., 2020; Knights et al., 2021).

Transcranial direct-current stimulation (tDCS) has been identified as an effective and non-invasive strategy to modulate brain function (Nitsche and Paulus, 2000; Borchers et al., 2011; Kunze et al., 2016; Mancini et al., 2016) and subsequently also behavior (Jacobson et al., 2012). One major field of tDCS research is the stimulation of motor-relevant brain areas and its effects on motor skill learning and performance (Reis and Fritsch, 2011; Ammann et al., 2016; Buch et al., 2017), pointing toward the fact that tDCS might be able to support maintenance or even restoration of motor dexterity. Indeed, research indicates that motor dexterity can be facilitated using tDCS over the primary motor cortex M1, (Boggio et al., 2006; Sohn et al., 2012; Karok and Witney, 2013; Kidgell et al., 2013; Convento et al., 2014). In old adults, improvements in motor performance were found *via* concurrent stimulation over the M1 contralateral to the trained hand (Hummel et al., 2010; Goodwill et al., 2013; Zimerman et al., 2013) even though other studies only found improvements in case of non-dominant M1 stimulation (Marquez et al., 2015). Furthermore, M1 tDCS affects retention of motor learning suggesting that tDCS concurrently applied with motor practice supports older adults to retain their improvements in performance (Parikh and Cole, 2014).

It is known that even simple unimanual motor tasks induce bilateral increases in cortical excitability (Cramer et al., 1999; L. Lee et al., 2003; Koeneke et al., 2006; Carroll et al., 2008), presumably due to hemispheric specialization in controlling certain movement features like movement speed, acceleration, movement duration or final position information (Sainburg and Wang, 2002; Schaefer et al., 2009). However, even though each hemisphere may be related to certain distinct features, information are transferred between hemispheres (Schaefer et al., 2009). The ability to perform a motor task with the opposite, untrained limb after learning with the other limb is a phenomenon known as cross-limb transfer (van Mier and Petersen, 2006; Koeneke et al., 2009; Stockel and Wang, 2011; Pan and Van Gemmert, 2013). In previous studies, mainly M1 ipsilateral to the trained limb was investigated with regard to its contribution in cross-limb transfer (Bodwell et al., 2003; M. Lee et al., 2010). In older adults, anodal tDCS (a-tDCS) applied over the M1 ipsilateral to the trained limb facilitated cross-limb transfer and increased motor overflow in the untrained hand (Goodwill et al., 2015). Since motor overflow is associated with increased bi-hemispheric activity (Bodwell et al., 2003), cross-limb transfer effects were mainly attributed to the increase in untrained hemisphere activation induced by tDCS. However, greater bilateral activation during unimanual motor tasks does not necessarily manifest in more pronounced cross-limb transfer (Hinder et al., 2011). Furthermore, other studies found cross-limb transfer impairments after enhancement of M1 ipsilateral to the trained limb (Keitel et al., 2018) and others even found cross-limb transfer enhancement after suppression of the ipsilateral M1 (Kobayashi et al., 2009). Thus, it might be possible that other mechanisms than bi-hemispheric activation are responsible for cross-limb transfer improvements. Therefore, finding evidence on how tDCS over M1 contralateral to the trained limb affects cross-limb transfer in a unimanual motor task would be of great importance. Besides, studying cross-limb transfer as a mechanism to improve fine motor performance is of high practical relevance. For some old adults, active hand training is impossible due to disease or injury and even in healthy aging, cross-limb transfer effects could be used for example to align asymmetrical fine motor abilities. Furthermore, using a neuromodulatory tool such as tDCS during motor task performance may help compensating age-related reductions in brain function (Hummel et al., 2010; Cabeza et al., 2018) and thereby modulate neuroplasticity (Zimerman and Hummel, 2010). On a more general note, finding strategies to maintain or restore fine motor function aids to keep old people an active part of society since fine motor abilities are an important prerequisite of independent living (Ranganathan et al., 2001b; Choi et al., 2017).

In the current study, we aimed at assessing the effect of a single a-tDCS session over right M1 while older adults performed the grooved pegboard test (GPT) with their left hand (LH_*trained*_). Cross-limb transfer was investigated in the right, untrained hand (RH_*untrained*_) before and after LH training. The GPT was previously verified as a reliable measure of motor dexterity (Desrosiers et al., 1995) and has been attributed to general cognitive functioning in older age (Ashendorf et al., 2009). Motor performance measures of LH_*trained*_ and RH_*untrained*_ included three recovery measurements 15, 30 and 45 min after LH training to assess possible tDCS after-effects, which were previously found within 1 h after termination of the stimulation (Koo et al., 2016) and can be regarded as markers of ongoing neuroplastic processes (Koo et al., 2016). We hypothesized that motor performance of the RH_*untrained*_ improves after a-tDCS over right M1 and that improvements outlast the stimulation period, pointing toward the capability of tDCS contralateral to the trained limb to persistently facilitate cross-limb transfer. Furthermore, we hypothesized that in line with previous studies, a-tDCS over right M1 also facilitates motor performance of LH_*trained*_ (Hummel et al., 2010; Goodwill et al., 2013; Zimerman et al., 2013; Parikh and Cole, 2014).

## Materials and methods

### Ethical approval

This study was approved by the local ethics committee of Leipzig University (ref. nr. 202-13-150-72013). All participants provided written informed consent and all procedures were conducted in accordance with the Declaration of Helsinki.

### Participants

An *a priori* sample size calculation (G*Power 3.1) specified a total sample size of 22 participants to obtain a moderate effect size *f* = 0.25 and power of 1-β = 0.8 using a mixed model with two groups and five measurement points and a significance level of α < 0.05. A moderate effect size was assumed since a recent meta-analysis (Summers et al., 2016) found a robust medium effect (standardized mean difference = 0.65) for the modulation of motor task performance in older adults using tDCS. To ensure high enough sample size also in cases of drop outs, we enrolled a total of 32 volunteers (16 female). Participants were included according to the following criteria: (a) age between 60 and 80 years, (b) a score of at least 24 on the Mini-Mental State Examination (MMSE), and (c) right-handedness according to the Edinburgh Handedness Inventory (Oldfield, 1971). The exclusion criteria were as follows: (a) cognitive impairments, (b) focal cortical lesions, (c) neurological or psychiatric disorders, (d) drug abuse, (e) previous neurosurgical operations, (f) migraine, (g) epilepsy or other cerebral seizures, (h) a pacemaker, (i) intracranial metal clips (j) cochlear implants, (k) liquor-shunt, (l) pregnancy, (m) sleep disorders, or (n) brain activity influencing medication. All participants were neurologically assessed by a qualified physician before the experiment. Following this examination, one female participant was excluded because of migraine and one male participant because of a strong manual tremor. Finally, 30 healthy adults (15 female, 15 male; age: 67.0 ± 4.6 years (mean ± standard deviation); laterality quotient (LQ): 93.7 ± 11.7) were included in the experiment. Participants were randomly assigned to one tDCS-stimulation group (anodal tDCS (a-tDCS); *n* = 15) or a sham-group (s-tDCS; *n* = 15). The number of hours of physical exercise and video gaming during an average week was assessed with a questionnaire to control for potential confounding factors on manual dexterity. Additionally, the amount of sleep (h) before the experiment was assessed. To assess baseline levels of attention, the d2-R test (Brickenkamp et al., 2010) was performed before the experiment. Here, the parameter of accuracy (total of crossed-out items divided by errors) was used as the main outcome measure. An overview of participant characteristics is provided in Table 1. All participants received monetary compensation for their participation.

**TABLE 1:** Overview of participant characteristics for both groups (a-tDCS and s-tDCS).

Variable	tDCS-group (a-tDCS)	Sham-group (s-tDCS)	*P*-value Mann–Whitney
Sample size	*n* = 15	*n* = 15	−
Gender (f/m)	5/10	10/5	−
Age (years)	66.5 ± 4.7	67.6 ± 4.7	0.453
Handedness (LQ)	90.8 ± 15.0	96.6 ± 6.3	0.311
Physical activity (h/wk)	2.6 ± 2.5	2.8 ± 2.0	0.477
Video games (h/wk)	1.1 ± 2.5	1.8 ± 2.8	0.414
Time slept (h)	7.8 ± 1.1	7.5 ± 1.2	0.464
d2-R accuracy	139.5 ± 22.7	154.3 ± 28.7	0.191

All values are expressed as mean ± standard deviation. Group differences were tested with pairwise Mann–Whitney U tests.

### Experimental procedure

**FIGURE 1:**
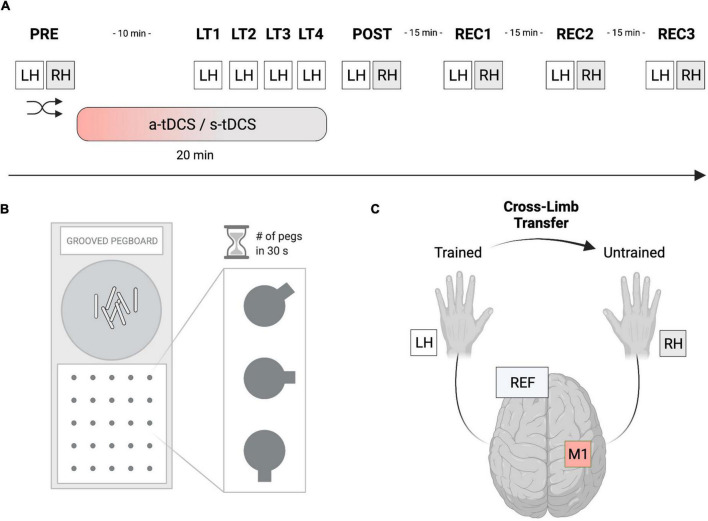
Experimental setup. **(A)** In this randomized, double-blinded, sham-controlled study, participants had to perform a manual dexterity task called the Grooved Pegboard Test (GPT). The baseline performance of both hands was assessed (PRE), before performance of the left hand (LH_trained_) was investigated during 4 learning trials (LT1-LT4). Thereafter, performance improvements of both hands were reassessed (POST). Furthermore, 3 recovery trials (REC1-REC3) were performed, separated by 15 min of rest to assess tDCS after-effects. Anodal transcranial direct current stimulation (a-tDCS) or sham stimulation (s-tDCS) was applied for 20 min during motor practice of LH_trained_. The order of left- or right-hand testing was randomized per timepoint. **(B)** The Grooved Pegboard Test (GPT) was implemented to investigate eye-hand coordination and motor speed for manual dexterity. **(C)** During a-tDCS and s-tDCS, LH_trained_ performed the GPT while RH_untrained_ was resting. Anodal tDCS of the right M1 was applied for 20 min with the reference electrode placed on the left supraorbital cortex. Created with Biorender.com.

In this randomized, double-blinded, sham-controlled study (see Figure 1A), participants had to perform a manual dexterity task called the grooved pegboard test (GPT). Thereafter, GPT was completed once, as a baseline measure, with each hand (left hand: LH; right hand: RH) before tDCS was applied to the right M1 (PRE) but with the tDCS-electrodes already attached to the scalp. Next, a-tDCS was applied for a total duration of 20 min and was initiated 10 min before participants started training GPT performance. The training consisted of a total of 4 learning trials (LT1-LT4) with the left (non-dominant) hand (LH_*trained*_) including rest phases of 90 seconds in between each successive trial. After a-tDCS or s-tDCS, GPT performance of both hands (POST) was immediately re-assessed. Additionally, 3 recovery trials (REC1, REC2, REC3) with 15-min breaks between each of the trials were performed to assess tDCS after-effects. The order of LH or RH trials during PRE, POST, REC1, REC2, and REC3 were randomized. Before and after tDCS, a Visual Analogue Scale (VAS) was used to measure participants’ level of attention, tiredness, and pain (scale from 1 to 10). All experiments were performed in a laboratory environment with an ambient temperature between 19 and 22°C.

### Manual dexterity task—grooved pegboard test

The GPT (Model 32025, Lafayette Instrument, United States) was used to measure eye-hand coordination and motor speed for manual dexterity (see Figure 1B). The test consists of a board with a matrix of five-by-five holes, each containing a groove pointing in a specific direction. In contrast to the Purdue Pegboard Test, where the holes are round, participants had to rotate the metal pins called Pegs to fit into the grooves of the board matrix. This design added another level of complexity to the manual task requiring not only motor abilities but also visuomotor coordination. For each trial, the number of pegs participants were able to correctly put into the respective slot was measured within 30 s as the outcome measure of interest. Participants always started on the upper left-hand side of the matrix continuing on the row to the right side before switching to a lower row. The trial time of 30 s was measured with a stopwatch and only fully completed pegs were counted. In the starting position, the participants sat comfortably, with the pegboard aligned straight in front of them and both hands resting next to it. The pegs were picked up by the participant from the intended area of the board (see Figure 1).

### Transcranial direct current stimulation

Transcranial direct current stimulation (tDCS) was applied *via* a battery-driven stimulator (neuroConn GmbH, Ilmenau, Germany) with two attached electrodes (see Figure 1C). The active (anodal) electrode had a size of 5 × 5 cm while an electrode size of 10 × 10 cm was chosen for the reference (cathodal) electrode. According to the 10–20 system, the active electrode was positioned over the right M1 contralateral to LH_*trained*_ (electrode position C4). The reference electrode was placed on the contralateral (left) supraorbital cortex. The scalp was first rubbed with alcohol, then both electrodes were soaked in saline and fixed to the scalp with rubber bands. A-tDCS of 1 mA was applied for 20 min with a fade-in and fade-out period of 30 s each. During s-tDCS, the current was ramped up for 30 s, held constant at 1 mA for 30 s, and ramped down for 30 s. This short duration of stimulation has been shown to elicit no changes in cortical excitability while it may provide the same tingling sensation on the scalp of the participant (Nitsche et al., 2008). Electrode montage, active electrode size (5 × 5 cm) and current intensity were essentially adopted from a previous study (Goodwill et al., 2015). However, a larger reference electrode was used to minimize the influence of cathodal frontal cortex stimulation (Kaminski et al., 2013). Generally, the impedance was monitored and kept under 10 Ω. Both the researcher and the participants were blinded.

### Statistical analysis

All statistical analyses were performed using JASP (Version 0.16, JASP Team 2021). The majority of the GPT performance variables were normally distributed according to Shapiro–Wilk-Tests (α = 0.05). Demographic and handedness variables, d2-R accuracy as well as other questionnaire variables (VAS) were not normally distributed. To compare these variables between a-tDCS and s-tDCS, non-parametric Mann–Whitney *U* tests were used.

Baseline GPT performance of both hands (LH_*trained*_ and RH_*untrained*_) was compared between groups (a-tDCS, s-tDCS) using independent samples *t*-tests. Subsequently, all individual GPT values obtained at POST, REC1, REC2, and REC3 were normalized to values obtained at PRE. This normalization procedure was done separately for each hand. Furthermore, individual motor learning trials (LT1-LT4) were also normalized to baseline GPT performance of LH_*trained*_ obtained during PRE.

GPT performance of LH_*trained*_ was assessed using a repeated-measures ANOVA with the within-subject factor TRIAL (PRE, LT1, LT2, LT3, LT4, POST), the between-subject factor GROUP (a-tDCS, s-tDCS) and sex of the participants as covariate.

GPT performance during recovery trials and cross-limb transfer effects to RH_*untrained*_ was assessed using repeated-measures ANOVAs with the within-subject factor TRIAL (PRE, POST, REC1, REC2, REC3), the between-subject factor GROUP (a-tDCS, s-tDCS) and sex as covariate for each hand separately.

VAS ratings of attention, tiredness, and pain before and after stimulation were compared within groups using Wilcoxon-signed rank tests. Additionally, VAS ratings were compared between groups (a-tDCS, s-tDCS) using separate Mann–Whitney *U* tests.

The statistical threshold was set at *p* = 0.05 for all analyses. For ANOVAs, partial eta squared (η_*p*_^2^) was used to report effect sizes while Cohen’s d was used for Bonferroni corrected *post hoc* comparisons. Effect sizes for the pairwise Mann–Whitney *U* test were expressed with the rank-biserial correlation coefficient (r). When the sphericity assumption of repeated-measures ANOVAs was violated, a Greenhouse-Geisser correction was applied.

## Results

### Participant characteristics

Participants of the randomly allocated groups a-tDCS and s-tDCS did not differ in terms of age (*W* = 131.00, *p* = 0.453, *r* = 0.164), LQ (*W* = 133.50, *p* = 0.311, *r* = 0.186), the amount of physical activity (*W* = 130.00, *p* = 0.477, *r* = 0.156) or videogaming (*W* = 129.50, *p* = 0.414, *r* = 0.151) during an average week and the amount of sleep before the experiment (*W* = 94.50, *p* = 0.464, *r* = −0.160) (see Table 1). Furthermore, no difference in attention before the experiment as assessed by d2-R accuracy could be revealed between groups (*W* = 144.50, *p* = 0.191, *r* = 0.284).

### Baseline grooved pegboard test performance at PRE

Baseline GPT performance of LH_*trained*_ did not differ between group (a-tDCS: 9.6 pegs; s-tDCS: 8.9 pegs; t_(28)_ = 1.134, *p* = 0.266, *d* = 0.414). However, for RH_*untrained*_, baseline GPT performance was slightly higher in s-tDCS compared to a-tDCS (a-tDCS: 9.9 pegs, s-tDCS: 11.5 pegs; t_(28)_ = −2.112, *p* = 0.044, *d* = −0.771; see Table 2).

**TABLE 2:** Overview of raw Grooved Pegboard Test performance (number of pegs) and percentage improvement (normalized to values obtained at PRE) for both groups (a-tDCS, s-tDCS) for LH_trained_ and RH_untrained_.

	a-tDCS				s-tDCS			
		
	LH_trained_		RH_untrained_		LH_trained_		RH_untrained_	
				
	Pegs	%	Pegs	%	Pegs	%	Pegs	%
PRE	9.6 ± 1.7	100	9.9 ± 2.0	100	8.9 ± 1.5	100	11.5 ± 2.0	100
POST	11.1 ± 1.8	117.3	11.5 ± 1.2	118.7	10.8 ± 1.4	122.7	11.8 ± 2.0	103.5
REC1	10.7 ± 1.7	112.7	11.3 ± 1.6	117.1	10.9 ± 1.3	123.3	11.8 ± 1.9	103.8
REC2	10.8 ± 2.1	113.3	11.4 ± 1.8	118.0	11.3 ± 2.0	127.2	12.1 ± 1.8	106.2
REC3	11.5 ± 1.6	121.2	11.8 ± 1.6	121.7	10.8 ± 1.5	122.6	12.0 ± 1.4	106.1

All values are expressed as mean ± standard deviation.

### Manual dexterity motor performance

**FIGURE 2:**
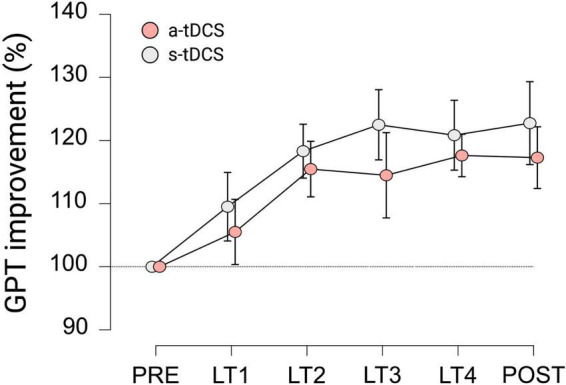
Motor performance of LH_trained_ during learning trials (LT) of GPT for both groups. All values are normalized to values obtained at PRE, representing percentage improvements. Displayed are mean ± 95% confidence intervals.

With regard to GPT performance of LH_*trained*_, repeated measures ANOVA indicated a significant effect for TRIAL (F_(3.525, 95.186)_ = 9.623, *p* < 0.001, η_*p*_^2^ = 0.263). Pairwise *post hoc* comparisons revealed, that for both groups, GPT performance during LT2 (mean difference (MD) = 16.9%, *p* < 0.001, *d* = 1.124), LT3 (MD = 18.5%, *p* < 0.001, *d* = 1.231), LT4 (MD = 19.2%, *p* < 0.001, *d* = 1.280) and POST (MD = 20.0%, *p* < 0.001, *d* = 1.332) was significantly higher compared to baseline performance during PRE (see Figure 2). However, no significant effect for GROUP (F_(1, 27)_ = 0.651, *p* = 0.318, η_*p*_^2^ = 0.012) and no significant interaction effect GROUP × TRIAL (F_(3.525, 95.186)_ = 0.240, *p* = 0.896, η_*p*_^2^ = 0.009) was observed.

### Performance during recovery and cross-limb transfer effects

**FIGURE 3:**
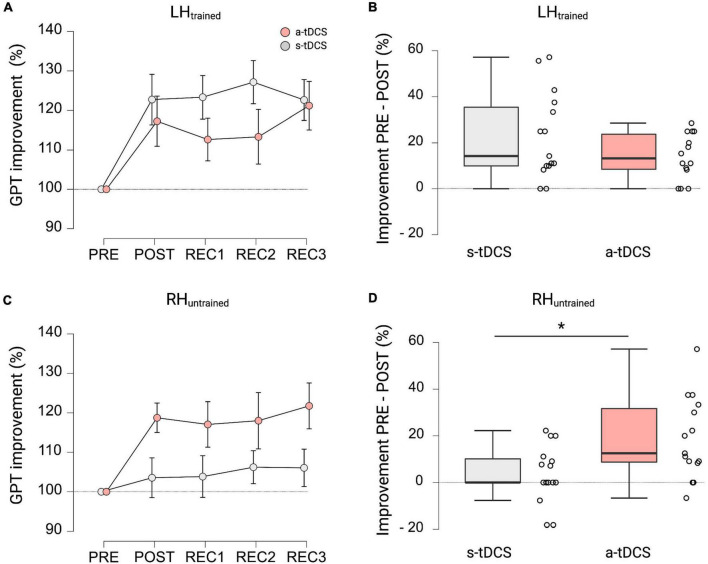
GPT performance during recovery trials and cross-limb transfer for both groups. **(A)** Motor performance of LH_trained_. Displayed are mean ± 95% confidence intervals. All values are normalized to values obtained at PRE, representing percentage improvements. **(B)** Percentage improvements in GPT of LH_trained_ from PRE to POST between a-tDCS and s-tDCS. No significant difference in motor performance was observed between groups. **(C)** Cross-limb transfer of RH_untrained_. Displayed are mean ± 95% confidence intervals. All values are normalized to values obtained at PRE, representing percentage improvements. **(D)** Percentage improvements in GPT of RH_untrained_ from PRE to POST between a-tDCS and s-tDCS. A-tDCS demonstrated significantly higher cross-limb transfer compared to s-tDCS. *Indicates a significant group difference.

In terms of GPT performance of LH_*trained*_ during recovery trials, repeated measures ANOVA revealed a significant effect for TRIAL (F_(4, 108)_ = 10.182, *p* < 0.001, η_*p*_^2^ = 0.274). Pairwise *post hoc* tests indicated that both groups improved GPT performance of LH_*trained*_ during POST (MD = 20.0%, *p* < 0.001, *d* = 1.247), REC1 (MD = 18.0%, *p* < 0.003, *d* = 1.121), REC2 (MD = 20.3%, *p* < 0.001, *d* = 1.262), and REC3 (MD = 21.9%, *p* < 0.001, *d* = 1.366) compared to baseline performance obtained during PRE (see Figure 3A). However, no significant effect for GROUP (F_(1, 27)_ = 1.487, *p* = 0.233, η_*p*_^2^ = 0.052; see Figure 3B) and no significant interaction effect GROUP × TRIAL (F_(4, 108)_ = 1.485, *p* = 0.212, η_*p*_^2^ = 0.052) was observed.

When looking at cross-limb transfer effects to RH_*untrained*_, repeated measures ANOVA revealed a significant effect for GROUP (F_(1, 27)_ = 4.234, *p* = 0.049, η_*p*_^2^ = 0.136) and TRIAL (F_(2.842, 76.728)_ = 3.030, *p* = 0.037, η_*p*_^2^ = 0.101). Pairwise *post hoc* comparisons for TRIAL showed that GPT performance of RH_*untrained*_ during POST (MD = 11.1%, *p* = 0.001, *d* = 0.724), REC1 (MD = 10.5%, *p* = 0.003, *d* = 0.679), REC2 (MD = 12.1%, *p* < 0.001, *d* = 0.787), and REC3 (MD = 13.9%, *p* < 0.001, *d* = 0.903) was significantly higher compared to baseline performance during PRE (see Figure 3C). The *post hoc* test for GROUP showed, that a-tDCS had higher improvements in GPT performance of RH_*untrained*_ and therefore higher cross-limb transfer compared to s-tDCS (MD = 10.6%, *p* < 0.049, *d* = 0.376; see Figure 3D). Again, the interaction effect GROUP × TRIAL failed to reach significance (F_(2.842, 86.728)_ = 2.275, *p* = 0.090, η_p_^2^ = 0.078).

### Attention, tiredness, pain (VAS scales)

No significant differences in levels of attention, tiredness, or pain could be detected between VAS ratings before and after stimulation for a-tDCS and s-tDCS (all *p* > 0.05). Furthermore, no significant between-group differences were observed for any of the VAS scales (all *p* > 0.05).

## Discussion

The main aim of the present study was to investigate whether a-tDCS applied over M1 contralateral to the trained limb is capable of enhancing cross-limb transfer in older adults. Furthermore, the effect of a-tDCS on motor dexterity performance in GPT with the non-dominant LH_trained_ was investigated. Our results showed that older adults improved GPT performance with their LH_trained_ and improvements persisted up to 45 min after training. Even more interestingly, older adults also improved RH_untrained_ performance but only when a-tDCS was applied during left hand training. RH_untrained_ improvement persisted during recovery measures, thus lasted for at least 45 min after training. After s-tDCS, no RH_untrained_ improvement was found, showing that cross-limb transfer was presumably mediated by modulation of right M1 excitability.

Our results support previous findings showing that older adults only exhibit cross-limb transfer after tDCS but not after sham stimulation (Goodwill et al., 2015). Our results show that stimulating M1 contralateral to the trained limb is capable of facilitating cross-limb transfer in older adults. This is of particular interest, since cross-limb transfer enhancement was previously mainly associated with increased activation in the hemisphere ipsilateral to the trained limb (Goodwill et al., 2015), hence due to increased bi-hemispheric activation. However, there is no causal relationship between the amount of bilateral activation and the amount of cross-limb transfer (Hinder et al., 2011) and other studies found divergent results after stimulating M1 ipsilateral to the trained limb (Kobayashi et al., 2009; Keitel et al., 2018). In our study, activation in the trained hemisphere was upregulated by a-tDCS, presumably resulting in reduced activation in the other (Tazoe et al., 2014) since M1-tDCS was shown to modulate remote interconnected neuronal networks apart from local M1 modulations in excitation and/or inhibition (Boros et al., 2008; Polania et al., 2011). Thus, alterations in interhemispheric communication can be regarded as one candidate mechanism responsible for cross-limb transfer improvements. There are different theories on how communication between hemispheres arises during unimanual motor tasks. While according to the cross activation hypothesis, bilateral activation induced by unimanual training evokes adaptations on both hemispheres, the bilateral access hypothesis states that information are resided at a central site other than the involved cortices (such as premotor cortex or supplementary motor area) where information can be accessed also from the untrained hemisphere (M. Lee et al., 2010). Our results are not exclusively in favor of one of the theories but do support the assumption that facilitated access to M1 contralateral to the trained limb enhances cross-limb transfer, suggesting a bilateral access either from the untrained M1 itself or from areas upstream of M1 (Boros et al., 2008).

It has been shown that dominant hand practice of GPT results in improved GPT performance of the non-dominant hand (Beg et al., 2021), speaking in favor of cross-limb transfer of the manual dexterity motor skill. The results of the current study expand these findings by showing that non-dominant hand training of GPT does *not* facilitate dominant hand performance in older adults receiving s-tDCS, suggesting that no skill transfer to the untrained hand took place. This result is in line with previous findings, showing that non-dominant hand performance improvements in most cases do not transfer to the dominant hand (Teixeira, 2000; Hinder et al., 2011). Differences between dominant and non-dominant hand transfer have mainly been attributed to more accurate internal models created by dominant hand training and better accessibility of the left (dominant) hemisphere (Wang and Sainburg, 2004; Pan and Van Gemmert, 2013). This explanation is also in line with our results where upregulation of the non-dominant right hemisphere by means of a-tDCS facilitated cross-limb transfer. In contrast to other studies (Kidgell et al., 2013; Marquez et al., 2015), a-tDCS did not facilitate left hand GPT learning. This finding was surprising since we expected tDCS over the right M1 to facilitate motor practice of the left hand especially because stimulation parameters comparable with other studies such as 20 min of stimulation duration, 1 mA current intensity and also similar electrode positions were used (Hummel et al., 2010; Marquez et al., 2015; Patel et al., 2019). However, GPT training only included four 30-second trials of left hand practice, thus may have been too short for the induction of differential tDCS effects. It might be, that longer training periods with a higher number of trials are necessary to observe differential GPT performance effects induced by tDCS. Besides, tDCS effects are highly variable across as well as within subjects and may be influenced by many different factors like age, sex, time of the day, quality of sleep or physical activity (Ridding and Ziemann, 2010; Polania et al., 2018). However, since a tDCS-induced effect on cross-limb transfer was found, we assume that most participants have responded to the stimulation. Interestingly, the missing tDCS effect on GPT performance of LH_trained_ suggests, that it is not the magnitude of training-induced improvement *per se* that is transferred *via* cross-limb transfer. Thus, a linear relationship between motor performance and cross-limb transfer is not necessarily evident (Goodwill et al., 2015) and both potentially evolve from different neural mechanisms. As postulated earlier, better accessibility of the non-dominant M1 induced by tDCS might be held responsible for facilitated cross-limb transfer, speaking in favor of the bilateral access hypothesis (Boros et al., 2008). Facilitation of GPT performance in trained limbs by means of tDCS, however, might need a sufficient time of concurrent M1-activation *via* both stimulation and training (Reis et al., 2008), which in our setup was potentially too short. Remarkably, we found that GPT performance improvement of the right hand induced by cross-limb skill transfer was comparable to GPT performance improvement of the left hand induced by training (13–20% skill improvement, see Table 2 for details). Transferred to absolute values, this means an improvement of around 1.5 pegs in 30 s, which seems like a small change. However, we never the less argue that this improvement is meaningful. Normative data states, that mean peg rate decreases by about 1 peg per decade (Agnew et al., 1988; Desrosiers et al., 1995). Given that this small but steady linear decrease is accompanied by major decreases in general cognitive functioning (Ashendorf et al., 2009), it is reasonable to assume that a 1.5 pegs improvement is relevant for daily living. This is also supported by a study showing that a difference of 1–2 pegs has high predictive value for a subsequent Parkinson’s diagnosis (Adler et al., 2002). Thus, one can conclude that cross-limb transfer may be a powerful way to improve performance in the opposite limb even in the absence of active practice. This may be of particular importance in the context of rehabilitation, where in some cases active training is impossible. One example could be either disease-related functional losses of hand motor function for example after stroke or Parkinson but also if active hand training is limited through limb pain or immobilization. Also, for healthy old adults, maximizing cross-limb transfer effects by using neuromodulatory tDCS could be important for example to align asymmetrical fine motor abilities.

The current study faces some limitations. Since only behavioral data was assessed and no control region was stimulated by means of a-tDCS, we can only speculate that the current findings were related to altered intra-/and/or interhemispheric communication. Furthermore, it would be of great importance to include also other stimulation sites such as the premotor cortex since it is known to be heavily involved in motor preparation and performance (Boros et al., 2008; Pavlova et al., 2022) and investigate potential mediatory effects on magnitude but also timing of transfer. Moreover, we found baseline differences between groups showing higher baseline GPT performance in the right hand in the sham group. However, all data was normalized to this first baseline timepoint and subsequent analyses were only performed using this normalized data. Furthermore, given that normative data shows, that older adults in the age range of our participants (60–69 years) are able to plug 13.9/13.1 pegs with their non-dominant hand in 30 s (Agnew et al., 1988; Desrosiers et al., 1995), we believe that possible ceiling effects of performance improvement did not affect our results. We are confident, that even though these baseline differences exist at least in one hand, performance improvements can nonetheless be interpreted. Furthermore, our groups contained an uneven number of male and female participants (s-tDCS: 10 females, a-tDCS: 5 females). Given that there is evidence for a strong effect of sex on simulated current intensity at older ages (Bhattacharjee et al., 2022), one could speculate that tDCS application created different brain activation patterns in our male and female participants. We addressed this issue by adding sex as a covariate to our statistical model, however, possible sex differences regarding responsiveness to tDCS may still have influenced our results.

Taken together, our study shows that tDCS over right M1 is capable of facilitating cross-limb transfer from left to right hand in older adults. Remarkably, the amount of cross-limb transfer induced by tDCS was comparable to the amount of skill learning induced by active training. This finding may specifically important in the context of neurorehabilitation, where training regimes other than active training are necessary to improve motor dexterity function.

## Data availability statement

The raw data supporting the conclusions of this article will be made available by the authors, without undue reservation.

## Ethics statement

The studies involving human participants were reviewed and approved by the Local Ethics Committee of Leipzig University (ref. nr. 202-13-150-72013), Stephanstraße 9a, 04103 Leipzig. The patients/participants provided their written informed consent to participate in this study.

## Author contributions

PB and PR designed the study. PB and MO collected the data. TM analyzed the data and created the figures. TM and EK drafted the manuscript. PB, MO, AV, and PR provided critical revision. All authors approved the final version of the manuscript and agreed to be accountable for all aspects of the work in ensuring that questions related to the accuracy or integrity of any part of the work are appropriately investigated and resolved.
